# Pulmonary Embolism Post-Bioprosthetic Aortic Valve Replacement: A Case Report

**DOI:** 10.1155/cric/7135717

**Published:** 2025-11-10

**Authors:** Hani Abdelaziz, Lydia-Dawn Tullak, Tania Levesque, Brent Wilkins, Borislav Bojilov, Muriel Berle, Steeve Landry, Riad Benghida, Mohamed Nashed

**Affiliations:** ^1^Pharmacy Department, Campbellton Regional Hospital, Vitalité Health, Campbellton, Canada; ^2^Medical Department, Campbellton Regional Hospital, Vitalité Health, Campbellton, Canada; ^3^Radiology Department, Campbellton Regional Hospital, Vitalité Health, Campbellton, Canada

**Keywords:** aortic valve, bioprosthetic, pulmonary embolism, replacement, thromboembolic

## Abstract

**Abstract:**

Pulmonary embolism is a rare complication post-bioprosthetic valve replacement, which is seldom reported in the literature.

**Case Description:**

A 76-year-old woman was admitted to our institution with a diagnosis of pulmonary embolism, occurring 14 days after undergoing a second bioprosthetic valve replacement, which had been performed due to failure of the initial prosthesis. Postoperatively, the patient had been managed with aspirin monotherapy for the prevention of thromboembolic events. Transthoracic echocardiography demonstrated normal age-related diastolic function, a left ventricular ejection fraction of 59%, no evidence of bioprosthetic valve stenosis, and overall satisfactory prosthetic valve function. The patient was discharged on the eighth hospital day with the initiation of warfarin therapy.

**Importance to Practitioners:**

It is crucial to closely monitor for clinical signs and symptoms of thromboembolism, such as pulmonary embolism, following bioprosthetic valve implantation, particularly in patients with high-risk factors including advanced age, female sex, hypertension, and a history of previous valve implantation failure. In such cases, clinicians should also consider intensifying antiplatelet or anticoagulation therapy beyond standard daily low-dose aspirin.

## 1. Introduction

Recent guidelines for valve replacements recommend using a vitamin K antagonist (VKA) in patients with mechanical heart valves or in patients with bioprosthetic valves with other indications requiring anticoagulation [1].

Bioprosthetic heart valves without underlying conditions requiring anticoagulation are not considered to be of sufficiently high risk to warrant anticoagulation or even dual antiplatelet (DAPT). [2] Despite this, multiple risk factors could increase the risk of thromboembolism associated with bioprosthetic valves. In these cases, the type of thromboembolism prophylaxis may be considered on a case-by-case basis [3].

## 2. Case Presentation

A 76-year-old woman presented with a 12-day history of progressive shortness of breath, which acutely worsened over the preceding 3–4 days to dyspnea at rest, palpitations, and left-sided chest discomfort. Her medical history included hypertension, osteoporosis, and two sequential bioprosthetic AVRs within 1 month. The first valve, implanted 33 days prior to admission, exhibited early structural dysfunction with echocardiographic evidence of moderate intraprosthetic regurgitation and severe stenosis. Although no definitive cause was identified, the prosthesis was returned to the manufacturer for further investigation due to a suspected structural or manufacturing defect. This prompted a second surgical bioprosthetic valve replacement, performed 14 days after the first. A postoperative transthoracic echocardiogram, conducted 6 days after the second procedure, confirmed a hemodynamically stable prosthesis with no evidence of obstruction or regurgitation.

**Figure 1:**
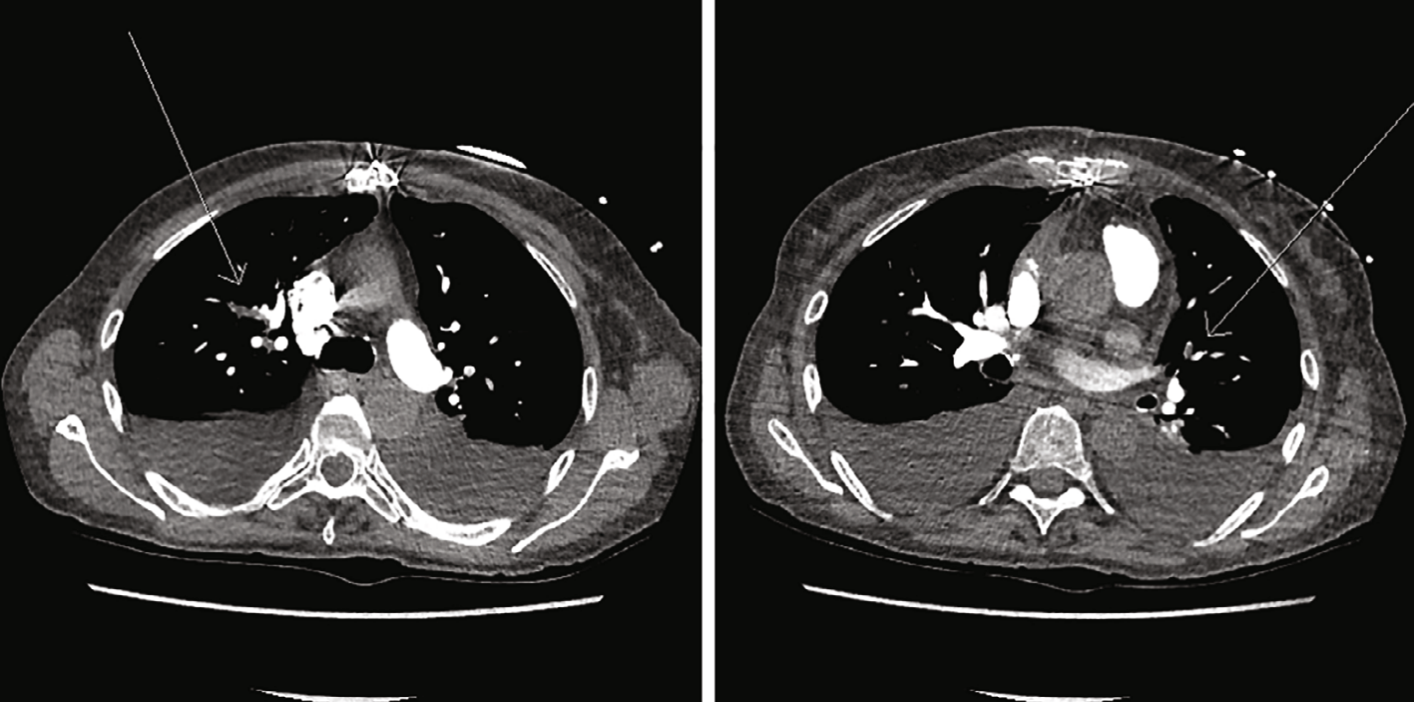
CT angiogram of the chest showing bilateral pulmonary emboli.

At the time of admission, the patient's home medications included metoprolol 50 mg twice daily, furosemide 40 mg daily, potassium chloride 20 mEq daily, acetylsalicylic acid (ASA) 81 mg daily, calcium 500 mg daily, risedronate 35 mg once weekly, pantoprazole 40 mg daily, docusate 200 mg twice daily, acetaminophen 500 mg as needed every 6 h, melatonin 3 mg at bedtime, cholecalciferol 10,000 IU once weekly, and oxazepam 15 mg as needed at bedtime. Computed tomography pulmonary angiography (CTPA) demonstrated multiple bilateral pulmonary emboli (Figure 1). On physical examination, no signs of systemic infection were noted throughout the hospitalization. A venous Doppler ultrasound of the lower extremities revealed no evidence of deep vein thrombosis (DVT); however, superficial thrombophlebitis involving the muscular and intermuscular tissues of the mid-left calf was identified.

On admission, the patient exhibited bradycardia with a blood pressure of 150/81 mmHg, heart rate of 47 bpm, respiratory rate of 22 breaths per minute, and oxygen saturation of 98% on room air. An initial electrocardiogram (ECG) confirmed sinus bradycardia. Then, 4 h later, repeat ECG revealed sinus tachycardia with frequent ventricular premature complexes (VPCs) occurring in runs of three or more, accompanied by a decrease in blood pressure to 110/71 mmHg, heart rate of 111 bpm, respiratory rate of 30, and oxygen saturation of 96% on room air. The rhythm progressed to atrial fibrillation with rapid ventricular response (*heart* *rate* > 99 bpm), along with transient ST-segment depression and episodes of ventricular bigeminy. The arrhythmia spontaneously converted to sinus rhythm, though intermittent irregularity persisted until hospital Day 4. Thereafter, the rhythm remained stable through discharge. Baseline laboratory results revealed a serum potassium of 5.0 mmol/L, C-reactive protein (CRP) of 37.5 mg/L, elevated troponin levels of 15.5 and 14.1 ng/L, and a B-type natriuretic peptide (BNP) of 3490 pg/mL. At discharge, vital signs were as follows: temperature 36.8°C, heart rate 82 bpm, respiratory rate 20, blood pressure 140/82 mmHg, and oxygen saturation 94% on room air.

Therapeutic anticoagulation was initiated on admission with concurrent subcutaneous dalteparin at 200 units/kg once daily and oral warfarin, following a standard bridging protocol. The patient remained hemodynamically stable and showed no signs of bleeding during hospitalization. By the time of discharge, the international normalized ratio (INR) was within the therapeutic range at 2.8. Clinically, the patient demonstrated significant improvement: She was independently ambulatory, with only mild exertional dyspnea, and pulmonary examination revealed clear lung fields. A repeat transthoracic echocardiogram showed preserved left ventricular ejection fraction (59%), normal age-related diastolic function, no evidence of prosthetic valve stenosis, and satisfactory bioprosthetic valve function. She was discharged on hospital Day 8 on her home medications, with the addition of warfarin.

## 3. Discussion

Complications associated with cardiac surgeries include the development of thromboembolic events [4]. In patients undergoing bioprosthetic valve implantation, multiple studies have highlighted the increased risk of stroke in the early postoperative period [5–9]. However, there is limited literature specifically addressing the risk of venous thromboembolism (VTE) in this setting. In addition to cardiac surgery itself, factors such as prosthetic valve implantation, the stented frame structure, and altered flow dynamics may further augment the thromboembolic risk [10, 11]. These mechanistic insights and evolving clinical observations underscore the need to clarify the optimal role and duration of anticoagulation following bioprosthetic valve replacement.

This clinical case underscores the potential for thromboembolic events following bioprosthetic AVR, even when guideline-based antiplatelet therapy is used. Our patient developed a pulmonary embolism while prescribed aspirin 81 mg daily after undergoing surgical bioprosthetic valve implantation. In a retrospective study, Heras et al. reported a 4% incidence of peripheral thromboembolism in patients who either did not receive anticoagulation or had subtherapeutic warfarin therapy during the first 90 days postoperatively, highlighting the importance of adequate early anticoagulation [12].

Despite experiencing some limitations in mobility following her second valve implantation, the patient was not bedridden or completely immobile, and venous Doppler studies were negative for DVT in the limbs. Therefore, we believe the pulmonary embolism was likely associated with the bioprosthetic valve implantation itself, potentially exacerbated by other contributing factors such as undergoing a second valve replacement, advanced age, and the absence of anticoagulation beyond aspirin monotherapy.

The safety and effectiveness of various thromboprophylaxis strategies following bioprosthetic valve replacement remain uncertain. A systematic review and meta-analysis comparing single and DAPT after transcatheter aortic valve replacement (TAVR) found no significant differences in all-cause mortality, cardiovascular death, stroke, or myocardial infarction; however, pulmonary embolism was not reported, and DAPT was associated with increased bleeding risk at 30 days [13]. A large cohort study involving 9060 veterans who underwent bioprosthetic AVR found no significant differences in thromboembolic outcomes among patients prescribed aspirin alone, warfarin alone, DAPT, or aspirin plus warfarin [14]. Similarly, an analysis of 25,656 nonveteran patients showed no difference in thromboembolic risk at 3 months between aspirin and warfarin groups [15].

Thrombosis Canada recommends long-term ASA 81 mg daily for patients with bioprosthetic AVR who have no other indication for anticoagulation [16].

Similarly, the American College of Cardiology (ACC) and the European Society of Cardiology (ESC) guidelines advocate for VKA therapy for 3 months or aspirin at a dosage of 75100 mg/day in patients with bioprosthetic valves who do not require long-term anticoagulation for other conditions, such as atrial fibrillation. In the setting of TAVR, the ACC guidelines suggest that short-term anticoagulation may be considered for the initial 36 months, followed by lifelong single antiplatelet therapy in patients without other indications for oral anticoagulation. The ESC guidelines recommend against the routine use of VKA after TAVI in patients without a baseline indication; however, in patients at increased risk of venous thromboembolism (VTE), such as our patient, VKA therapy should be considered [17, 18].

While small randomized controlled trials have failed to demonstrate a clear net clinical benefit of anticoagulation following bioprosthetic AVR [19, 20], observational evidence suggests otherwise. A large Danish registry reported that extended use of VKAs for up to 6 months postoperatively was associated with reduced rates of stroke and mortality, without a significant increase in bleeding events [11].

Moreover, emerging data highlight a greater-than-anticipated incidence of leaflet thrombosis in bioprosthetic valves after surgical implantation [21], reinforcing the rationale for early anticoagulation. When anticoagulation is indicated, initiation during the immediate postoperative period is recommended. Intravenous unfractionated heparin (UFH), titrated to achieve an activated partial thromboplastin time (aPTT) of 1.5–2 times the control value, facilitates rapid anticoagulant effect before therapeutic INR levels are achieved [22].

Given the heightened thromboembolic risk during the first postoperative month, timely initiation of anticoagulation is essential. While adjunctive aspirin may confer additional protection, its routine use alongside anticoagulants is not recommended due to elevated bleeding risk [23]. In patients with low bleeding risk, current guidance supports VKA therapy targeting an INR of 2.5 for a minimum of 3 months, and potentially up to 6 months, following surgical bioprosthetic valve implantation [18].

The GALILEO Trial found that in patients undergoing TAVR with no indication for long-term anticoagulation, rivaroxaban was associated with higher rates of thromboembolism, mortality, and bleeding. As a result, the study was prematurely terminated [24]. However, a substudy found that rivaroxaban resulted in a lower incidence of subclinical leaflet thrombosis observed via a four-dimensional CT scan compared to antiplatelet therapy [25]. The results from this substudy may encourage future studies with dosing optimization of DOACs post-TAVR.

The ENAVLE trial evaluated 220 patients who underwent surgical aortic or mitral valve replacement or repair and received edoxaban (30 mg or 60 mg once daily) or warfarin for 3 months. Edoxaban was found to be noninferior to warfarin with respect to composite endpoints, including thrombotic complications [26]. Therefore, the role of DOACs in preventing thromboembolism still needs to be evaluated in future studies, such as the upcoming Atlantis Trial evaluating apixaban post-TAVR [27].

## 4. Conclusion

Our patient underwent a second bioprosthetic valve implantation due to the failure of the first valve, and aspirin 81 mg/day was prescribed for its antiplatelet effect. She subsequently experienced a pulmonary embolism with a brief period of atrial fibrillation, which was anticoagulated with warfarin. Considering these events and the presence of other risk factors, such as older age, female sex, and hypertension, the patient is at increased risk of thromboembolic events. Although current guidelines recommend single antiplatelet therapy following TAVR in standard-risk patients, for those with additional thromboembolic risk factors, an initial regimen for reducing the risk of thromboembolism could include either DAPT or anticoagulation with warfarin. Further consideration of performing a four-dimensional CT scan in patients with a failed valve replacement may help identify subclinical leaflet thrombosis and require anticoagulation with a DOAC, such as rivaroxaban.

## Data Availability

The authors have nothing to report.

## References

[1] Macle L., Cairns J. A., Andrade J. G. (2015). The 2014 Atrial Fibrillation Guidelines Companion: A Practical Approach to the Use of the Canadian Cardiovascular Society Guidelines. *Canadian Journal of Cardiology*.

[2] Blair K. L., Hatton A. C., White W. D. (1994). Comparison of Anticoagulation Regimens After Carpentier-Edwards Aortic or Mitral Valve Replacement. *Circulation*.

[3] Gonzalez-Lavin L., Tandon A. P., Chi S. (1984). The Risk of Thromboembolism and Hemorrhage Following Mitral Valve replacement. *Journal of Thoracic and Cardiovascular Surgery*.

[4] Gillinov A. M., Davis E. A., Alberg A. J., Rykiel M., Gardner T. J., Cameron D. E. (1992). Pulmonary Embolism in the Cardiac Surgical Patient. *Annals of Thoracic Surgery*.

[5] Vranckx P., Windecker S., Welsh R. C., Valgimigli M., Mehran R., Dangas G. (2017). Thrombo-Embolic Prevention After Transcatheter Aortic Valve Implantation. *European Heart Journal*.

[6] Huygens S. A., Mokhles M. M., Hanif M. (2016). Contemporary Outcomes After Surgical Aortic Valve Replacement With Bioprostheses and Allografts: A Systematic Review and Meta-Analysis. *European Journal of Cardio-Thoracic Surgery*.

[7] Rashid H., Gooley R., Thein P. (2018). Bioprosthetic Aortic Valve Leaflet Thrombosis Detected by Multidetector Computed Tomography Is Associated With Adverse Cerebrovascular Events: A Meta-Analysis of Observational Studies. *Heart, Lung & Circulation*.

[8] Breyne J., Juthier F., Corseaux D. (2010). Atherosclerotic-Like Process in Aortic Stenosis: Activation of the Tissue Factor–Thrombin Pathway and Potential Role Through Osteopontin Alteration. *Atherosclerosis*.

[9] Marechaux S., Corseaux D., Vincentelli A. (2009). Identification of Tissue Factor in Experimental Aortic Valve Sclerosis. *Cardiovascular Pathology*.

[10] Hasanoğlu C., Argüder E., Kılıç H., Parlak E. S., Karalezli A. (2019). Atrial Fibrillation, an Obscured Cause of Pulmonary Embolism Can Be Revealed by Adding to Wells Criteria. *Clinical Research*.

[11] Mérie C., Køber L., Olsen P. S. (2012). Association of Warfarin Therapy Duration After Bioprosthetic Aortic Valve Replacement With Risk of Mortality, Thromboembolic Complications, and Bleeding. *JAMA*.

[12] Heras M., Chesebro J. H., Fuster V. (1995). High Risk of Thromboemboli Early After Bioprosthetic Cardiac Valve Replacement. *Journal of the American College of Cardiology*.

[13] Vavuranakis M., Siasos G., Zografos T. (2016). Dual Or Single Antiplatelet Therapy After Transcatheter Aortic Valve Implantation? A Systematic Review and Meta-Analysis. *Current Pharmaceutical Design*.

[14] Bravata D. M., Coffing J. M., Kansagara D. (2019). Association Between Antithrombotic Medication Use After Bioprosthetic Aortic Valve Replacement and Outcomes in the Veterans Health Administration System. *JAMA Surgery*.

[15] Brennan J. M., Edwards F. H., Zhao Y. (2012). Early Anticoagulation of Bioprosthetic Aortic Valves in Older Patients: Results From the Society of Thoracic Surgeons Adult Cardiac Surgery National Database. *Journal of the American College of Cardiology*.

[16] Thrombosis Canada (2024). *Mechanical and Bioprosthetic Heart Valves: Anticoagulant Therapy [Clinical Guide on the Internet]*.

[17] Otto C. M., Nishimura R. A., Bonow R. O. (2021). 2020 ACC/AHA Guideline for the Management of Patients With Valvular Heart Disease: A Report of the American College of Cardiology/American Heart Association Joint Committee on Clinical Practice Guidelines. *Circulation*.

[18] Praz F., Borger M. A., Lanz J. (2025). 2025 ESC/EACTS Guidelines for the management of valvular heart disease: Developed by the task force for the management of valvular heart disease of the European Society of Cardiology (ESC) and the European Association for Cardio-Thoracic Surgery (EACTS). *European Journal of Cardio-Thoracic*.

[19] Sundt T. M., Zehr K. J., Dearani J. A. (2005). Is Early Anticoagulation With Warfarin Necessary After Bioprosthetic Aortic Valve Replacement?. *Journal of Thoracic and Cardiovascular Surgery*.

[20] ElBardissi A. W., DiBardino D. J., Chen F. Y., Yamashita M. H., Cohn L. H. (2010). Is Early Antithrombotic Therapy Necessary in Patients With Bioprosthetic Aortic Valves in Normal Sinus Rhythm?. *Journal of Thoracic and Cardiovascular Surgery*.

[21] Egbe A. C., Pislaru S. V., Pellikka P. A. (2015). Bioprosthetic Valve Thrombosis Versus Structural Failure: Clinical and Echocardiographic Predictors. *Journal of the American College of Cardiology*.

[22] Iung B., Rodes-Cabau J. (2014). The Optimal Management of Anti-Thrombotic Therapy After Valve Replacement: Certainties and Uncertainties. *European Heart Journal*.

[23] Laffort P., Roudaut R., Roques X. (2000). Early and Long-Term (One-Year) Effects of the Association of Aspirin and Oral Anticoagulant on Thrombi and Morbidity After Replacement of the Mitral Valve With the St. Jude Medical Prosthesis: A Clinical and Transesophageal Echocardiographic Study. *Journal of the American College of Cardiology*.

[24] Dangas G. D., Tijssen J. G., Wöhrle J. (2020). A Controlled Trial of Rivaroxaban After Transcatheter Aortic-Valve Replacement. *New England Journal of Medicine*.

[25] De Backer O., Dangas G. D., Jilaihawi H. (2020). Reduced Leaflet Motion After Transcatheter Aortic-Valve Replacement. *New England Journal of Medicine*.

[26] Shim C. Y., Seo J., Kim Y. J. (2023). Efficacy and Safety of Edoxaban in Patients Early After Surgical Bioprosthetic Valve Implantation or Valve Repair: A Randomized Clinical Trial. *Journal of Thoracic and Cardiovascular Surgery*.

[27] Montalescot G., Redheuil A., Vincent F. (2022). Apixaban and Valve Thrombosis After Transcatheter Aortic Valve Replacement: The ATLANTIS-4D-CT Randomized Clinical Trial Substudy. *Cardiovascular Interventions*.

